# Supplementary material for: Pre-clinical evaluation of biomarkers for the early detection of nephrotoxicity following alpha-particle radioligand therapy
Source: Eur J Nucl Med Mol Imaging. 2023 Dec 14;51(5):1395–408. doi: 10.1007/s00259-023-06559-9 (PMC10957612; doi:10.1007/s00259-023-06559-9)
Supplement: Supplementary file 1 — Supplementary file1 (PDF 2.09 MB) [file 259_2023_6559_MOESM1_ESM.pdf]

Pre-clinical Evaluation of Biomarkers for the Early Detection of Nephrotoxicity Following Alpha-particle Radioligand Therapy

European Journal of Nuclear Medicine and Molecular Imaging

Mengshi Li,[1*] Claudia Robles-Planells,[2*] Dijie Liu,[1] Stephen A. Graves,[3] Gabriela Vasquez-Martinez,[2] Gabriel Mayoral-Andrade,[2] Dongyoul Lee,[4] Prerna Rastogi,[5] Brenna M. Marks,[1] Edwin A. Sagastume,[1] Robert M. Weiss,[6] Sarah C. Linn-Peirano,[2,7] Frances L. Johnson,[1] Michael K. Schultz,[1,3,8#] Diana Zepeda-Orozco[2,9,10#]

[1]Viewpoint Molecular Targeting, Inc., Coralville, IA, USA

[2]Kidney and Urinary Tract Center, Abigail Wexner Research Institute at Nationwide Children's, Columbus, Ohio, USA

[3]Department of Radiology, The University of Iowa, Iowa City, IA, USA.

[4]Department of Physics and Chemistry, Korea Military Academy, Seoul, Republic of Korea

[5]Department of Pathology, The University of Iowa, Iowa City, IA, USA

[6]Department of Internal Medicine, The University of Iowa, Iowa City, IA, USA

[7]Department of Veterinary Biosciences, The Ohio State University College of Veterinary Medicine Columbus, OH

[8]Department of Pediatrics, The Ohio State University College of Medicine, Columbus, Ohio, USA.

[9]Division of Nephrology and Hypertension, Nationwide Children's Hospital, Columbus, Ohio, USA.

[10]Department of Radiation Oncology, Free Radical and Radiation Biology Program, The University of Iowa, Iowa City, IA, USA.

[*]Co-First Authors

[#]Co-Corresponsing Authors

Schultz, Michael: mischult@uiowa.edu

Diana Zepeda-Orozco: Diana.zepeda-orozco@nationwidechildrens.org

Supplemental Table 1. Scoring Criteria for Kidney Histological Changes

Tubular Injury Scoring		Glomerular Changes	
0	Absent	0	Absent
1	Mild (1-10%)	1	1-10%
2	Moderate (11-25%)	2	11-20%
3	Severe (26-50%)	3	21-30%
4	Very Severe > 50%	4	31% and greater

Tubulointerstitial Inflammation		Interstitial Fibrosis	
0	Absent	0	Absent
1	Mild (1-10%)	1	Mild (1-10%)
2	Moderate (11-25%)	2	Moderate (11-25%)
3	Severe (26-50%)	3	Severe (26-50%)
4	Very Severe > 50%	4	Very Severe > 50%

Supplemental Table 2. Ex vivo [^{203}Pb]Pb-MC1L biodistribution results for male CD-1 Elite mice (Units of percent injected dose per gram, corrected for [^{203}Pb] radioactive decay).

**[^{203}Pb]Pb-MC1L Percent injected dose per gram (%ID/g)**					
**Tissue**	**0.5 h**	**1.5 h**	**3.0 h**	**24 h**	**Avg. Organ Mass (g)**
**Blood**	1.37	0.06	0.01	0.02	3.000*
**Heart**	0.68	0.03	0.03	0.06	0.132
**Lungs**	2.15	0.19	0.12	0.08	0.173
**Liver**	0.64	0.40	0.49	0.32	1.409
**Pancreas**	0.50	0.04	0.13	0.57	0.023
**Spleen**	0.63	0.22	0.19	0.21	0.101
**Stomach**	0.86	0.08	0.11	0.06	0.423
**Kidneys**	8.30	4.88	4.51	1.99	0.467
**Adrenals**	1.31	0.34	0.27	0.42	0.011
**Small Intestines**	0.64	0.06	0.07	0.03	1.334
**Large Intestine**	0.86	0.06	0.10	0.06	1.379
**Testes**	0.41	0.07	0.03	0.05	0.183
**Muscle**	0.34	0.18	0.06	0.06	11.250**
**Skin**	1.12	0.22	0.12	0.13	5.625***
**Bone**	0.46	0.11	0.09	0.14	0.069
**Brain**	0.04	0.01	0.02	0.02	0.369
**Eyes**	0.40	0.08	0.23	0.12	0.032

* Assuming 8% of total body mass is blood.
** Assuming 30% of total body mass is muscle.
*** Assuming 15% of total body mass is skin.

Supplemental Table 3. Percent administered activity ([^{212}Pb]Pb-MC1L) per organ as a function of time, calculated from [^{203}Pb]Pb-MC1L ex vivo biodistribution data.

[^{212}Pb]Pb-MC1L percent administered activity per (%AA/organ)				
**Tissue**	**0.5 h**	**1.5 h**	**3.0 h**	**24 h**
**Blood**	3.978	0.163	0.025	0.013
**Heart**	0.086	0.004	0.003	0.002
**Lungs**	0.359	0.030	0.017	0.003
**Liver**	0.867	0.511	0.568	0.094
**Pancreas**	0.011	0.001	0.003	0.003
**Spleen**	0.061	0.020	0.016	0.004
**Stomach**	0.350	0.031	0.038	0.005
**Kidneys**	3.750	2.066	1.731	0.195
**Adrenals**	0.014	0.003	0.002	0.001
**Small Intestines**	0.830	0.073	0.077	0.008
**Large Intestine**	1.147	0.075	0.113	0.017
**Testes**	0.073	0.012	0.005	0.002
**Muscle**	3.659	1.836	0.555	0.141
**Skin**	6.098	1.122	0.555	0.153
**Bone**	0.031	0.007	0.005	0.002
**Brain**	0.016	0.003	0.006	0.002
**Eyes**	0.013	0.002	0.006	0.001

Supplemental Table 4. Bi-exponential fit parameters, and time-integrated activity coefficients for the two integration methods. $\tau 1$: Time-integrated activity coefficient calculated from the bi-exponential fit. $\tau 2$: time integrated activity coefficient calculated from trapezoidal integration assuming constant activity from 0 to 0.5 h, and physical decay beyond the terminal timepoint.

Fit coefficients:	A_1	λ_1	A_2	λ_2	τ_1	τ_2	τ_{avg}
**Blood**	21.164	3.356	0.026	0.029	0.0720	0.0478	0.0599
**Heart**	0.789	4.508	0.003	0.019	0.0034	0.0017	0.0026
**Lungs**	1.890	3.441	0.022	0.082	0.0081	0.0066	0.0074
**Liver**	12.640	7.763	0.063	0.072	0.0250	0.1033	0.0642
**Pancreas**	*	*	*	*	*	0.0011	0.0011
**Spleen**	0.178	2.878	0.019	0.066	0.0036	0.0038	0.0037
**Stomach**	2.598	4.226	0.037	0.068	0.0115	0.0096	0.0106
**Kidneys**	8.897	3.553	2.364	0.104	0.2524	0.3084	0.2804
**Adrenals**	0.043	2.555	0.002	0.033	0.0008	0.0007	0.0008
**Small Intestines**	5.745	4.067	0.080	0.078	0.0245	0.0200	0.0222
**Large Intestine**	11.872	4.853	0.101	0.060	0.0412	0.0297	0.0354
**Testes**	0.212	2.281	0.005	0.041	0.0022	0.0019	0.0021
**Muscle**	7.112	1.191	0.511	0.051	0.1600	0.1585	0.1593
**Skin**	17.542	2.330	0.645	0.060	0.1829	0.1771	0.1800
**Bone**	0.099	2.709	0.006	0.043	0.0017	0.0015	0.0016
**Brain**	0.243	6.130	0.005	0.031	0.0019	0.0013	0.0016
**Eyes**	0.462	7.928	0.004	0.046	0.0015	0.0011	0.0013

*Regression not well defined for Pancreas due to increase in estimated tissue concentration after 1.5 hours.

Supplemental Table 5. Summary of mouse and human organ masses and organ [212Pb]Pb-MC1L time-integrated activity coefficients.

Tissue	m_{mouse} (g)	τ_{mouse} (MBq*h/MBq)	m_{human} (g)	τ_{human} (MBq*h/MBq)
Blood	3.000	5.99E-02	6364	2.42E-01
Heart	0.132	2.55E-03	330	8.61E-03
Lungs	0.173	7.38E-03	1200	8.14E-02
Liver	1.409	6.42E-02	1800	1.01E-01
Pancreas	0.023	1.13E-03	140	3.46E-03
Spleen	0.101	3.67E-03	150	4.30E-03
Stomach	0.423	1.06E-02	250	8.56E-03
Kidneys	0.467	2.80E-01	310	3.22E-01
Adrenals	0.011	7.77E-04	14	8.00E-04
Small Intestines	1.334	2.22E-02	350	8.41E-03
Large Intestine	1.379	3.54E-02	225	8.54E-03
Testes	0.183	2.06E-03	35	4.51E-04
Muscle	11.250	1.59E-01	29200	4.24E-01
Skin	5.625	1.80E-01	3600	1.32E-01
Bone	0.069	1.58E-03	4400	8.61E-02
Brain	0.369	1.59E-03	1450	5.36E-03
Eyes	0.032	1.28E-03	15	6.86E-04

Supplemental Table 6. Murine absorbed dose estimates by tissue for [^{212}Pb]Pb-MC1L, generated using OLINDA 2.2.

	Dose per administered activity (mGy/MBq)			
**Tissue**	**Alpha**	**Beta**	**Gamma**	**Total**
**Brain**	2.05E+01	7.46E+00	4.14E-01	2.84E+01
**Large Intestine**	1.67E+02	2.14E+01	7.75E-01	1.89E+02
**Small Intestine**	1.26E+02	1.76E+01	1.40E+00	1.45E+02
**Stomach**	1.64E+02	1.94E+01	3.48E-01	1.84E+02
**Heart**	1.37E+02	2.03E+01	1.15E+00	1.59E+02
**Kidneys**	2.76E+03	2.34E+02	3.18E+00	2.99E+03
**Liver**	2.09E+02	2.87E+01	1.41E+00	2.39E+02
**Lungs**	1.99E+02	1.52E+01	5.18E-01	2.14E+02
**Pancreas**	2.16E+02	2.89E+02	9.81E+00	5.15E+02
**Skeleton**	1.29E+02	5.30E+01	6.23E+00	1.88E+02
**Spleen**	1.06E+02	2.10E+01	1.23E+00	1.29E+02
**Testes**	4.98E+01	9.81E+00	5.21E-01	6.01E+01
**Thyroid**	4.91E+01	5.68E+00	4.97E-01	5.53E+01
**Total Body**	4.91E+01	1.14E+01	4.87E-01	1.14E+02
**Blood***	8.98E+01	-	-	8.98E+01
**Adrenals***	3.13E+02	-	-	3.13E+02
**Muscle***	6.37E+01	-	-	6.37E+01
**Skin***	1.44E+02	-	-	1.44E+02
**Eyes***	1.78E+02	-	-	1.78E+02

* Blood, adrenals, muscle, skin, and eyes are not implemented as source/target tissues in the OLINDA 2.2 murine models, and therefore dose to these tissues was calculated assuming local alpha particle energy deposition, with an average alpha energy of 7.8063 MeV per [^{212}Pb] decay. Beta and gamma self-dose contributions were not calculated for these tissues.

Supplemental Table 7. Estimated human dosimetry for [^{212}Pb]Pb-MC1L.

Tissue	Alpha (mGy/MBq)	Beta (mGy/MBq)	Gamma (mGy/MBq)	Total (mGy/MBq)
**Adrenals**	2.62E-01	4.54E-02	3.26E-02	3.40E-01
**Brain**	1.69E-02	1.89E-03	2.07E-03	2.09E-02
**Esophagus**	4.70E-02	5.32E-03	7.24E-03	5.96E-02
**Eyes**	2.10E-01	2.17E-02	3.37E-03	2.35E-01
**Gallbladder**	4.70E-02	6.04E-03	1.14E-02	6.44E-02
**Left Colon**	4.70E-02	1.52E-02	9.92E-03	7.21E-02
**Small Intestine**	4.70E-02	1.16E-02	7.98E-03	6.65E-02
**Stomach**	4.70E-02	1.44E-02	8.87E-03	7.03E-02
**Right Colon**	4.70E-02	1.52E-02	8.96E-03	7.11E-02
**Rectum**	4.70E-02	5.29E-03	5.60E-03	5.78E-02
**Heart**	1.20E-01	1.36E-02	7.77E-03	1.41E-01
**Kidneys**	**4.76E+00**	**5.17E-01**	**5.91E-02**	**5.34E+00**
**Liver**	2.58E-01	2.90E-02	1.37E-02	3.01E-01
**Lungs**	3.11E-01	3.33E-02	7.02E-03	3.51E-01
**Pancreas**	1.13E-01	1.23E-02	1.03E-02	1.36E-01
**Prostate**	4.70E-02	5.29E-03	5.69E-03	5.79E-02
**Salivary**	4.70E-02	5.29E-03	3.60E-03	5.59E-02
**Red Marrow**	**2.43E-01**	**1.32E-02**	**6.39E-03**	**2.63E-01**
**Osteogenic Cells**	1.17E+00	2.60E-02	6.11E-03	1.20E+00
**Spleen**	1.31E-01	1.54E-02	1.38E-02	1.61E-01
**Testes**	5.91E-02	6.23E-03	3.50E-03	6.87E-02
**Thymus**	4.70E-02	5.41E-03	5.68E-03	5.80E-02
**Thyroid**	4.70E-02	5.29E-03	4.78E-03	5.71E-02
**Urinary Bladder Wall**	4.70E-02	5.29E-03	4.91E-03	5.72E-02
**Total Body**	8.84E-02	1.02E-02	4.66E-03	1.03E-01
